# Intrahepatic peribiliary perivascular epithelioid cell tumor (PEComa) associated with heterotopic pancreas: A case report

**DOI:** 10.1186/s13000-016-0528-9

**Published:** 2016-08-20

**Authors:** Yuka Kiriyama, Tetsuya Tsukamoto, Yoshikazu Mizoguchi, Shin Ishihara, Akihiko Horiguchi, Takamasa Tokoro, Yutaro Kato, Atsushi Sugioka, Makoto Kuroda

**Affiliations:** 1 Department of Diagnostic Pathology, Fujita Health University School of Medicine, 1-98 Dengakugakubo, Kutsukake-cho, Toyoake, Aichi 470-1192 Japan; 2 Department of Surgery, Fujita Health University School of Medicine, Toyoake, Aichi Japan

**Keywords:** Perivascular epithelioid cell tumor (PEComa), Liver, Heterotopic pancreas

## Abstract

**Background:**

Perivascular epithelioid-cell tumor (PEComa) is a group of rare mesenchymal neoplasms that express myomelanocytic-cell markers and exhibit a wide variety of histopathological features. Although heterotopic pancreas has been reported to occur in the gastrointestinal tract, intrahepatic heterotopic pancreas has been reported only rarely.

**Case presentation:**

We present a case of intrahepatic PEComa that showed a strong regional correlation with the presence of heterotopic pancreas. An intrahepatic tumor and biliary dilatation was incidentally discovered during a diagnostic evaluation to investigate low-back pain in a 47-year-old Japanese male. Cholangiocarcinoma was suspected and a left hemihepatectomy performed. Histological examination revealed a 3 × 3.8-mm tumor in the neighboring B2 bile duct. Histological and immunohistochemical investigations revealed the presence of a PEComa and pancreatic acini within the tumor mass. PEComa in the hepatobiliary and pancreatic regions are extremely rare. The presence of heterotopic pancreas is also relatively uncommon.

**Conclusion:**

The strong regional association of these 2 lesions raises the possibility of a PEComa originating from heterotopic pancreas or from an irritable response caused by heterotopic pancreas.

## Background

Perivascular epithelioid cell tumors (PEComas) constitute a rare family of mesenchymal tumors that can occur in any part of the human body. Cases developing in the liver are, however, extremely rare [1]. PEComas arising in the pancreas are even rarer, and only 12 cases have been reported so far [2]. Heterotopic pancreatic tissue is usually found in the gastrointestinal tract; however, it is rarely detected in the liver and only 0.5–13.7 % have been identified in autopsies [3]. To our knowledge, no cases of a combination of PEComa and heterotopic pancreas have been previously described. We present here an incidentally found case of intrahepatic PEComa that was strongly associated with heterotopic pancreas tissue.

## Case presentation

**Fig. 1:**
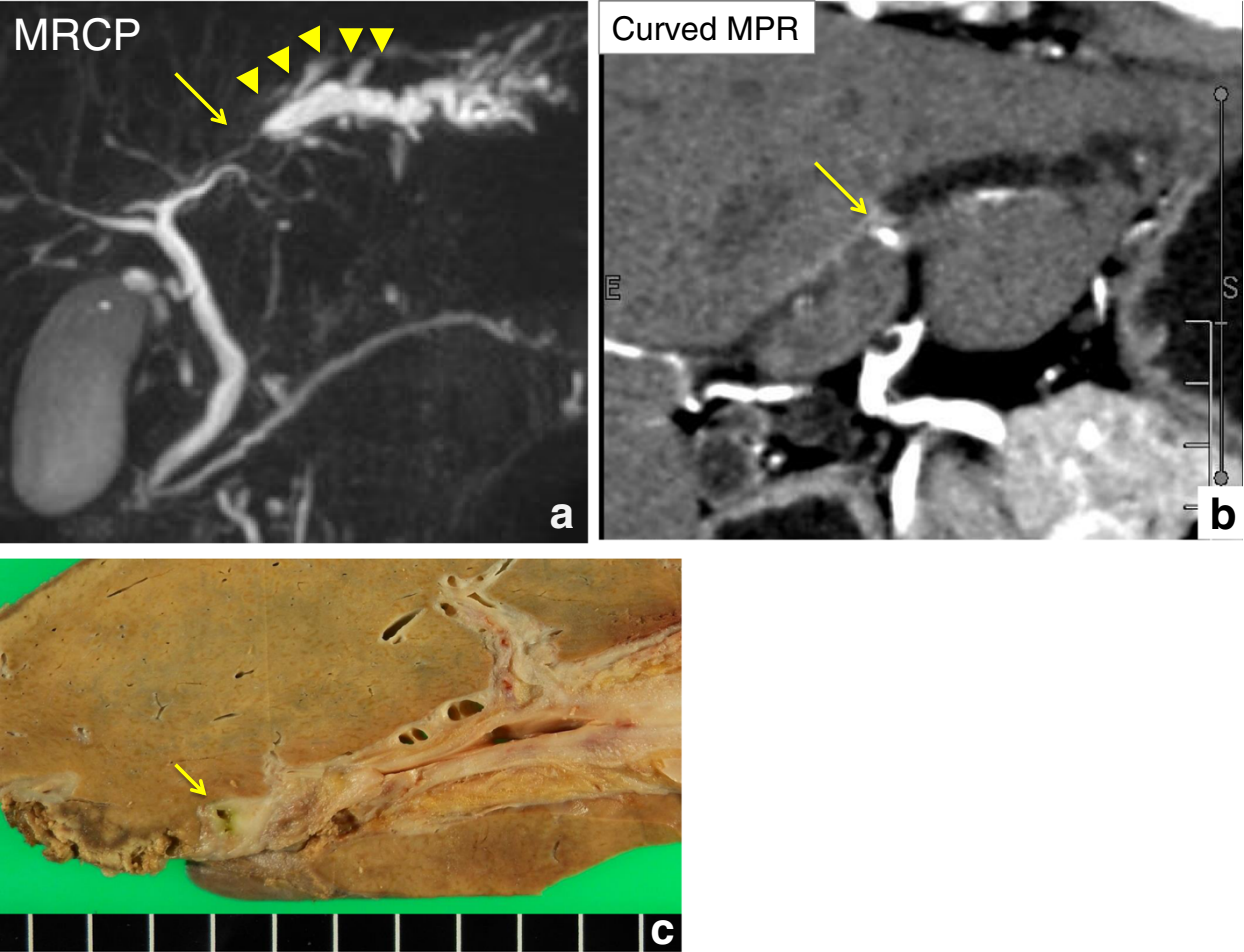
Radiological and macroscopic features of the bile duct and liver tumor. **a** Magnetic resonance cholangiopancreatography (MRCP). The B2 bile duct is obstructed (arrow) and dilated at its periphery (arrow heads). **b** Curved multiplanar reconstruction image of contrast-enhanced CT. The liver tumor is visualized as a high-intensity area in the arterial phase (arrow). **c** Macroscopic view of the tumor. Grayish tumor tissue is observed (arrow)

A 47-year-old Japanese man visited a local doctor complaining of low-back pain 5 months before admission to our hospital. The patient had no relevant past or family history. Abdominal computed tomography (CT) and ultrasonography (US) incidentally revealed a dilatation of the intrahepatic bile duct. The patient was referred to our hospital for further investigation. Endoscopic retrograde cholangiopancreatography (ERCP) detected an obstruction of the B2 intrahepatic bile duct and magnetic resonance cholangiopancreatography showed dilatation of its peripheral ducts. The use of curved multiplanar reconstruction (MPR) of contrast-enhanced CT imaging detected a 1.8 × 0.5 cm high-intensity mass in the arterial phase at the obstructed region of the B2 bile dust (Fig. 1a, and b). Brush cytology failed to collect any samples for analysis. Laboratory examinations showed normal liver function test results but the presence of the carcinoembryonic antigen and cancer antigen 19–9 (CA19-9) tumor markers. An intrahepatic cholangiocarcinoma was suspected and a left hemihepatectomy was performed.

**Fig. 2:**
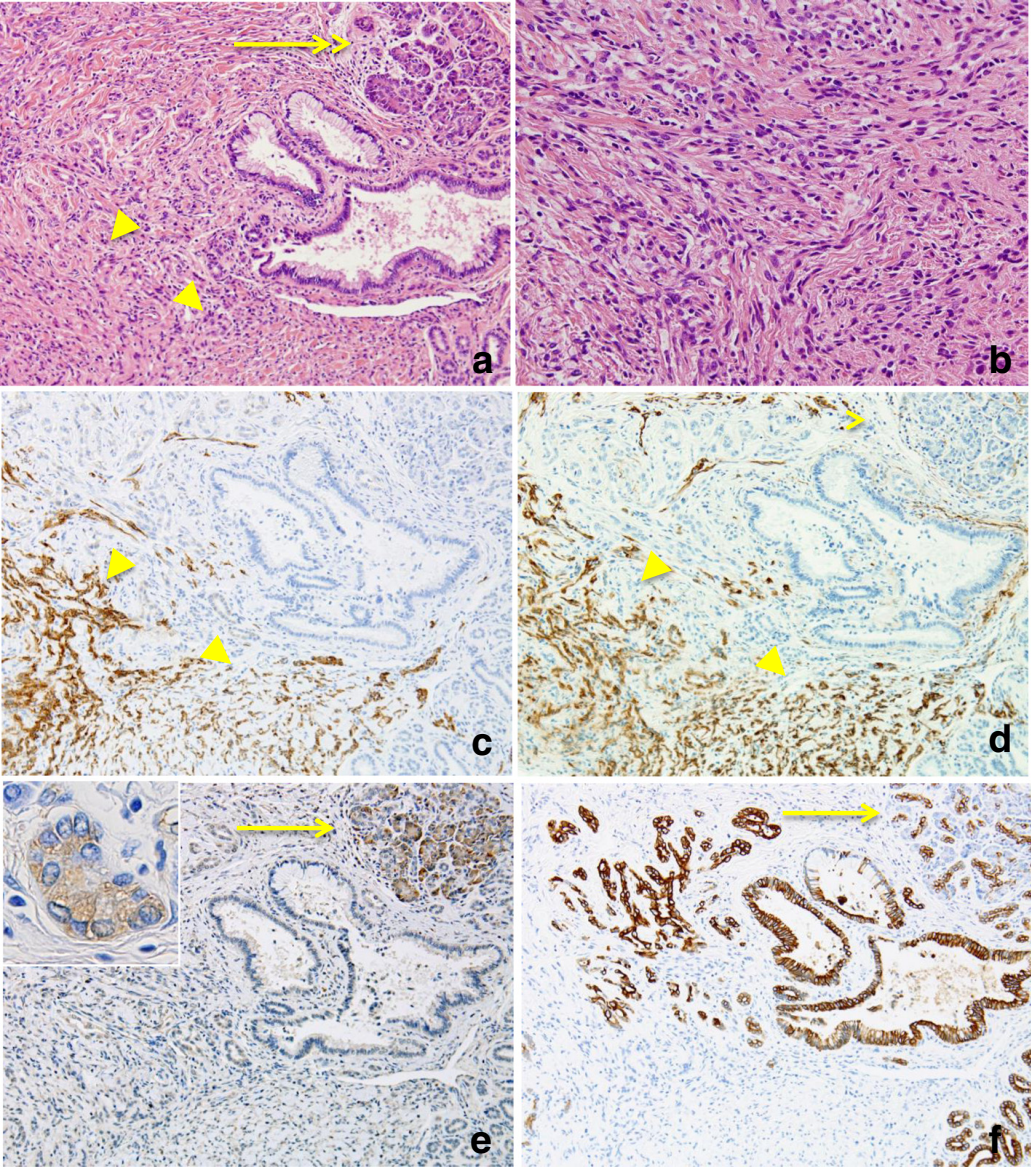
Histopathological and immunohistochemical features of heterotopic pancreas and PEComa. **a** Low-power view of the tumor, HE staining. **b** Higher magnification of PEComa, HE staining. **c** and **d** PEComa is immunoreactive for HMB-45 **c** and SMA **d. e** Heterotopic pancreatic acini are immunopositive for trypsin and α-amylase (inset). **f** Ducts are positive for CK19. Arrow: heterotopic pancreas, arrowhead: PEComa. Original magnification 100× **a**, **c**, **d**, **e**, and **f**, and 200× **b**

Macroscopically, the resected tissue specimen was a grayish mass of 3 × 3.8 mm with an ill-defined border but without a capsule (Fig. 1c). Routine hematoxylin and eosin (HE) stained sections from formalin-fixed, paraffin-embedded tissue were examined. Histologically, the tumor was composed of randomly arranged spindle cells with pale eosinophilic cytoplasm. Nuclear pleomorphism was not evident and mitotic figures were absent. Eosinophilic glands, resembling pancreatic acini and ductal structure, were observed in the periphery of the mass adjacent to the spindle tumor (Fig. 2a, and b).

Immunohistochemical studies showed a strong but diffuse distribution of the markers human melanoma black 45 (HMB45) and smooth muscle actin (SMA) in the spindle cells. These cells were negative for Melan-A, desmin, S-100, and cytokeratin 19 (CK19). The Ki-67 index was <1 %. The tumor was diagnosed as a PEComa (Fig. 2c, and d). Acinic lesions positive for trypsin but weakly positive for amylase-α were identified; they were considered as pancreatic acini. Ductal structures were CK19 positive, but were indistinguishable from either the pancreatic or intrahepatic biliary duct. Small ducts with the appearance of regenerative change were localized near the main biliary duct (Fig. 2e, and f). This lesion was considered to consist of heterotopic pancreas. Langerhans’ islets were not present. Overall the tumor was diagnosed as a PEComa associated with heterotopic pancreas. Biliary congestion was also apparent in the adjacent liver tissue. The patient was followed up for 56 months after surgery. No recurrence of the original tumor or metastasis has been observed.

## Discussion

We have documented an intrahepatic peribiliary tumor composed of the rare combination of PEComa and heterotopic pancreas. PEComa was characterized, both histologically and immunohistochemically, by the presence of distinctive perivascular epithelioid cells. The PEComa family includes a wide variety of different entities, such as angiomyolipoma (AML), lymphangioleiomyomatosis (LAM), and clear-cell “sugar” tumors (CCST) [4]. PEComas may also develop in various organ sites, including the liver, kidney, lung, pancreas, gastrointestinal tract, and soft tissue [4]. The presence of the HMB45 antigen has been found to be of value in the diagnosis of renal AML [5] as well as hepatic [6] and pulmonary [7] PEComas. In the current case, the tumor was composed of spindle cells expressing HMB45 and SMA, and it was diagnosed as a PEComa.

Hepatic PEComas occur principally in adults and predominantly in females [1]. Diagnosis of PEComas is, however, problematic. In a previous study, 9 patients incidentally identified as having asymptomatic liver tumors were preoperatively diagnosed on the basis of MRI and their clinical features as 6 hepatocellular carcinomas, 2 adenocarcinomas, and 1 AML [8]. In the current case, the tumor was initially considered an intrahepatic cholangiocarcinoma because of an obstruction of the B2 bile duct and dilatation of its periphery. PEComas vary widely in size ranging from 0.3 to >30 cm in diameter [9]. In 1 of the smallest cases of PEComa, an hepatic tumor of 0.8 cm was incidentally found during an operation for a gastric gastrointestinal stromal tumor (GIST) [10]. The case reported in the present study showed an early-stage tumor of only 3 × 3.8 mm. It is, therefore, difficult to make an accurate preoperative diagnosis of hepatic PEComa and to distinguish those with malignant potential from the benign lesions. Surgical resection with an adequate margin would, however, be the standard and appropriate procedure for the treatment of hepatic PEComa, because the majority of these tumors are benign [11].

Pancreatic PEComas are rare tumors. Twelve cases, with a predominance of tumors in females, have so far been reported [2]. Tumor sizes have ranged from 1.5 to 10 cm. The histopathological characteristics of the tumor cells arising from several organs are not distinguishable [12].

Folpe et al. [13] proposed classifying PEComas into 3 categories including benign, uncertain malignant potential, and malignant. They suggested that malignancy could be predicted by satisfying two or more of the following features: a >5-cm tumor, infiltrative growth, high nuclear grade and cellularity, the presence of >1 mitosis in 50 high-power fields, tumor necrosis, and vascular invasion. Because the present case did not fulfill any of these criteria, it was considered benign. Moreover, the patient has not shown any evidence of tumor recurrence or metastasis during the follow-up period for 56 months.

The presence of heterotopic pancreas in the hepatobiliary system [14] is rare compared with that in the gastrointestinal tract [15]. Indeed, 29 adenocarcinomas have been reported in heterotopic pancreas localized in gastrointestinal tracts [16]. Apart from the report by Yan et al. of an adenocarcinoma in intrahepatic heterotopic pancreas [17], no other tumors have been described as being associated with intrahepatic heterotopic pancreas so far.

## Conclusion

In conclusion, we report the extremely rare combination of an intrahepatic PEComa with a strong regional association with heterotopic pancreas. In the current case, the PEComa was possibly derived from the heterotopic pancreas or from the adjacent liver tissue. Further evaluation of tumorigenesis of the PEComa is warranted.

## Abbreviations

AML, angiomyolipoma; CA19-9, cancer antigen 19–9; CCST, clear-cell “sugar” tumors; CEA, carcinoembryonic antigen; CT, computed tomography; ERCP, endoscopic retrograde cholangiopancreatography; GIST, gastrointestinal stromal tumor; HE, hematoxylin and eosin; HMB45, human melanoma black 45; MPR, multiplanar reconstruction; PEComa, Perivascular epithelioid cell tumors; SMA, smooth muscle cell actin; US, ultrasonography
